# Scent of COVID-19: Whole-Genome Sequencing Analysis Reveals the Role of *ACE2*, *IFI44*, and *NDUFAF4* in Long-Lasting Olfactory Dysfunction

**DOI:** 10.3390/life15010056

**Published:** 2025-01-05

**Authors:** Beatrice Spedicati, Alessandro Pecori, Maria Pina Concas, Aurora Santin, Romina Ruberto, Giuseppe Giovanni Nardone, Andrea D’Alessandro, Giancarlo Tirelli, Paolo Boscolo-Rizzo, Giorgia Girotto

**Affiliations:** 1 Department of Medicine, Surgery and Health Sciences, University of Trieste, 34149 Trieste, Italy; beatrice.spedicati@burlo.trieste.it (B.S.); giuseppegiovanni.nardone@burlo.trieste.it (G.G.N.); andrea.dalessandro88@gmail.com (A.D.); tirellig@units.it (G.T.); paolo.boscolorizzo@units.it (P.B.-R.); giorgia.girotto@burlo.trieste.it (G.G.); 2 Institute for Maternal and Child Health, I.R.C.C.S. “Burlo Garofolo”, 34137 Trieste, Italy; alessandro.pecori@burlo.trieste.it (A.P.); aurora.santin@burlo.trieste.it (A.S.); romina.ruberto@burlo.trieste.it (R.R.)

**Keywords:** COVID-19, post-viral olfactory dysfunction, Whole Genome Sequencing, rare variants, common variants

## Abstract

COVID-19-related persistent olfactory dysfunction (OD) presents remarkable interindividual differences, and little is known about the host genetic factors that are involved in its etiopathogenesis. The goal of this study was to explore the genetic factors underpinning COVID-19-related OD through the analysis of Whole Genome Sequencing data of 153 affected subjects, focusing on genes involved in antiviral response regulation. An innovative approach was developed, namely the assessment of the association between a “gene score”, defined as the ratio of the number of homozygous alternative variants within the gene to its length, and participants’ olfactory function. The analysis highlighted how an increased gene score in the *ACE2* gene is associated with a worse olfactory performance, while an increased gene score in the *IFI44* and *NDUFAF4* genes is associated with a better olfactory function. Considering the physiological role of the proteins encoded by these genes, it can be hypothesized that a reduced expression of *ACE2* may be associated with a protracted and severe inflammatory response in the olfactory epithelium, thus worsening patients’ smell abilities. Conversely, an increased gene score in *IFI44* and *NDUFAF4* might be associated with a decreased inflammatory response, thus correlating with a better olfactory performance. Overall, this study identified new host genetic factors that may play a pivotal role in determining COVID-19-related OD heterogeneity, possibly enabling more personalized and effective clinical management for affected individuals.

## 1. Introduction

From an evolutionary perspective, the sense of smell is the most ancient sensory system, and it has long been crucial for the successful interaction between the individual and the environment [1]. Indeed, the correct identification of odorants has proven essential both for survival (i.e., recognition of potential poisons, spoiled foods, or smoke) and overall well-being (i.e., successful social relationships and mating) [2]. Therefore, olfactory dysfunction (OD) can have a significant impact on health and quality of life, and it has been shown that it may bring significant social, psychological, and emotional consequences, including depression, anxiety, lack of self-esteem and self-confidence, social isolation, and alterations in eating behavior [3]. Despite these considerable implications, olfactory dysfunction commonly goes unrecognized. In fact, with an overall population prevalence of OD of up to 24.5% [4], it has been estimated that fewer than 25% of patients are aware of their condition until they are psychophysically tested [5,6]. OD can be secondary to a number of factors, and, among all causes, upper respiratory tract viral infections are recognized as a major determinant of OD. Rhinoviruses, Adenoviruses, Influenza and Parainfluenza viruses, and Herpesviruses have long been known to be implicated in post-viral OD (PVOD) [7]. In recent years, PVOD has been gaining increasing attention from the medical community in consideration of its extraordinary high frequency among patients affected by Coronavirus disease 19 (COVID-19), caused by the Severe Acute Respiratory Syndrome Coronavirus 2 (SARS-CoV-2) [8,9]. Indeed, the global prevalence of OD in patients with COVID-19 has been estimated to be 47.85%, with significant differences according to geographical area, sex, and viral variant [10]. Several hypotheses have been formulated to explain OD in COVID-19, since, differently from what is seen in most upper respiratory tract viral infections, patients rarely present nasal congestion, and therefore conductive anosmia resulting from mechanical obstruction can be excluded [11]. In this light, sensorineural OD seems more plausible, and different mechanisms may be involved. In the olfactory epithelium (OE), the two proteins that are essential for viral entry, Angiotensin-Converting Enzyme 2 (ACE2) and transmembrane serine protease 2 (TMPRSS2), are abundantly expressed by olfactory neurons (ONs) supporting cells, i.e., sustentacular cells and Bowman gland cells, while they are not expressed by ONs themselves [12,13]. According to literature data, supporting cells alteration may justify the occurrence of OD during the acute phase of SARS-CoV-2 infection, but it does not explain why some patients experience long-lasting smell loss, which is defined as a persistence of the symptom more than 12 weeks after the infection [14]. Recently, the pathophysiology of persistent OD has been linked to the ability of SARS-CoV-2 to cause an extensive inflammatory response of the innate immune system in the OE, which can induce gene expression changes in the ONs [15].

Overall, the above-mentioned mechanism fails to explain why only a fraction of patients develops long-lasting olfactory alterations and why there is substantial variability in their onset, progression, and possible resolution. In these circumstances, host genetic factors may play a central role in interpreting these discrepancies, as they have already proven crucial to clarify interindividual variability in SARS-CoV-2 infection-related acute OD. Indeed, a multi-ancestry Genome-Wide Association Study (GWAS) has highlighted how polymorphisms in the *UGT2A1*/*UGT2A2* locus are associated with acute OD in patients with COVID-19 [16]. The *UGT2A1* gene encodes a glucuronosyltransferase involved in odorant metabolism that promotes the clearance of lipophilic molecules [17]; it is expressed by sustentacular cells, thus underlining once more the central role of supporting cells in acute anosmia pathogenesis. On the other hand, to date, very few works have explored the host genetic factors possibly associated with persistent OD [18]. Our study aims to fill this knowledge gap by applying an approach that has recently proven powerful in the identification of genes associated with the host response to COVID-19 [19,20,21], namely the analysis of the genetic landscape of rare and common variants. In particular, our study focuses on the identification of rare and common biallelic variants in genes involved in the antiviral response regulation pathway, taking advantage of Whole Genome Sequencing (WGS) data of a cohort of deeply characterized Italian patients who suffered from COVID-19 presenting with long-lasting OD.

## 2. Materials and Methods

### 2.1. Ethical Statement

This study was conducted in accordance with the Declaration of Helsinki and approved by the Ethics Committee of Friuli-Venezia Giulia Region (Application No. CEUR-2020-Os-156). Written informed consent was obtained from all participants for their participation in the study and for the collection of biological samples for research purposes.

### 2.2. Study Cohort and Clinical Evaluations

One-hundred and fifty-three adult patients aged 18 years or older, presenting with self-reported persistent OD due to mild COVID-19 and lasting over three months, were consecutively recruited by the Smell and Taste Outpatients Clinic of the Section of Otolaryngology of the University of Trieste (Trieste, Italy) between September 2021 and November 2022. Eligibility required a SARS-CoV-2 infection diagnosis confirmed via a Reverse Transcription Polymerase Chain Reaction (RT-PCR) test performed on a nasopharyngeal swab, following international guidelines [22]. Mild COVID-19 was defined by the presence of symptoms without lower respiratory disease evidence (clinical or imaging) and an oxygen saturation level of 94% or higher [23]. Standardized self-administered questionnaires were employed to collect basic demographic, lifestyle, and anamnestic data (i.e., age, sex, dates of first positive and negative COVID-19 tests, date of chemosensory alteration onset, information on comorbidities, such as hypertension, diabetes, chronic cardiovascular, liver, kidney, or respiratory disorders, and cancer). All patients underwent fiber-optic nasal endoscopy at enrollment. Individuals with previous head trauma, chronic rhinosinusitis, congenital anosmia, nasal polyps, or neurodegenerative disorders like Alzheimer’s or Parkinson’s disease were excluded from the study.

### 2.3. Psychophysical Assessment of Olfactory Function

Orthonasal olfactory function was measured using the extended Sniffin’ Sticks test (SST) battery (Burghart Messtechnik, Wedel, Germany), which includes the evaluation of odor thresholds (T), odor discrimination (D), and odor identification (I), as previously described [24,25]. The maximum score for each of the three subsections of the SST is 16. Results are combined for a composite TDI score (range 1–48) and categorized as anosmia (TDI ≤ 16), hyposmia (TDI between 16.25 and 30.5), or normosmia (TDI ≥ 30.75).

### 2.4. Biological Sample Collection and WGS Analysis

A peripheral whole blood sample was collected, and genomic DNA was extracted using the QIAsymphony^®^ SP instrument with the QIAsymphony^®^ Midi Kit (Qiagen, Venlo, The Netherlands), following the manufacturer’s instructions. DNA quality was verified with 1% agarose gel electrophoresis, and concentration was determined employing the Nanodrop ND 1000 spectrophotometer (NanoDrop Technologies Inc., Wilmington, DE, USA).

WGS was performed using the Illumina NovaSeq 6000 platform with the Illumina DNA Prep Kit (Illumina Inc., San Diego, CA, USA), according to the manufacturer’s protocol. After sequencing, FASTQ files were generated and analyzed through a standard pipeline. This pipeline comprises several steps, including (1) quality control performed with Fastqc software (https://www.bioinformatics.babraham.ac.uk/projects/fastqc/, last accessed on 19 July 2024) and Fastp software (v0.21.0) [26], retaining only reads with Q > 20, (2) sequence alignment to Human Genome Reference build 38 (GRCh38p.13) using BWA-mem (v2.1) [27], and (3) PCR duplicate removal and Base Quality Score Recalibration performed using, respectively, Sambamba (v1) [28] and GATK (v4.1.9.0) [29]. Joint variant calling was performed using a GATK haplotype caller and GenomicsDBImport [29], generating gVCF files. Afterwards, a quality check was performed applying Variant Quality Score Recalibration with GATK and the following filter thresholds: Hardy–Weinberg equilibrium (*p*-value < 10^−8^), missing rate (missing rate > 0.05), heterozygosity rate (*p*-value < 10^−8^), coverage (coverage pre variant calling ≥ 20), and singletons distribution (singletons distribution mean >3 standard deviations). Finally, data were phased using Eagle (v2.4.1) [30] and annotated using the Variant Effect Predictor (v106) [31].

### 2.5. Gene Selection

Coding variants that mapped within a list of 298 genes described in the literature as involved in antiviral response regulation were extracted from WGS data.

This list was created employing Ingenuity Pathway Analysis software (Ingenuity System Inc., Redwood City, CA, USA; https://digitalinsights.qiagen.com/products-overview/discovery-insights-portfolio/analysis-and-visualization/qiagen-ipa/, accessed on 23 September 2024) and then manually curated selecting only the genes described in the Online Catalog of Human Genes and Genetic Disorders (OMIM) classification (https://www.omim.org/, accessed on 23 September 2024). The complete list of the analyzed genes is reported in Table S1.

For each gene of the antiviral response list, the length was calculated extracting the position from BioMart (GRCh38.p12, Ensembl) [32].

### 2.6. Statistical Analysis

The following minor allele frequency (MAF) cutoffs were considered for the analysis and studied separately: (1) MAF ≤ 1%; (2) MAF between 1% and 5%; (3) MAF between 5% and 10%; and (4) MAF > 10%. Variants were coded 1 for alternative homozygotes and 0 for reference homozygotes and heterozygotes. For each individual and for each gene, a gene score was calculated, defined as the ratio of the number of homozygous alternative variants within the gene to its length in 100 kb.

For each gene, we tested the association between the gene score (independent variable) and orthonasal olfactory function tests results (dependent variables) with linear regression models. All models were corrected for sex and age. All analyses were performed on the entire cohort and on women and men separately; for X-linked genes, the analyses were performed only on women and men separately. All *p*-values were adjusted using the Benjamini–Hochberg method for each MAF group. An adjusted *p*-value of <0.05 was considered statistically significant. Statistical analyses were performed using the R software version 4.1.2 (R Foundation for Statistical Computing, Vienna, Austria).

## 3. Results

### 3.1. Demographic and Clinical Data

**Figure 1:**
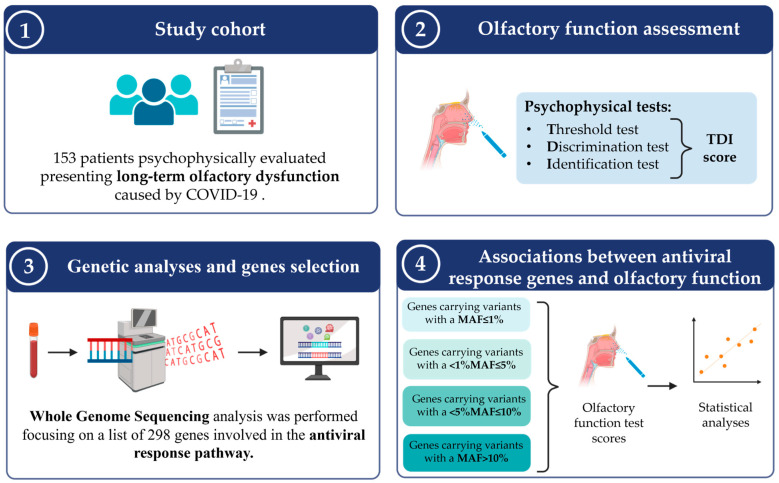
Study workflow. 1. A cohort of 153 patients presenting with persistent post-COVID-19 OD was selected. 2. Olfactory function was psychophysically assessed through the TDI score. 3. WGS analysis was performed, and biallelic variants in 298 genes involved in the antiviral response pathway were extracted. 4. Association analyses between gene score and olfactory performance were performed.

One hundred and fifty-three individuals affected by persistent OD after mild SARS-CoV-2 infection were enrolled in this study. All participants were of Caucasian origin, as confirmed by Principal Component Analysis. The complete study workflow is reported in Figure 1.

The mean age of the total cohort was 49.2 years, and the majority of the study sample was composed of female individuals (72.4%). In anamnesis, 13% of the patients, 14 women and five men, respectively, reported arterial hypertension, while 5.2% of the cohort was affected by cardiovascular diseases. Four women reported diabetes, and another female participant referred a tumor diagnosis. Finally, one female and two male participants were affected by a chronic respiratory disease. None of the enrolled subjects reported being affected by chronic liver or kidney disease (Table 1).

Each individual underwent a careful evaluation of the orthonasal olfactory function through the threshold, discrimination, and identification subtests; the results of the three tests were combined to obtain the overall TDI score. Complete results of the psychophysical tests in the total cohort stratified according to sex are reported in Table 2. Overall, 17% (26/153) of the total cohort were anosmic, 70.6% (108/153) were hyposmic, and, notably, 12.4% (19/153) obtained a final score within the normosmic range.

**Figure 2:**
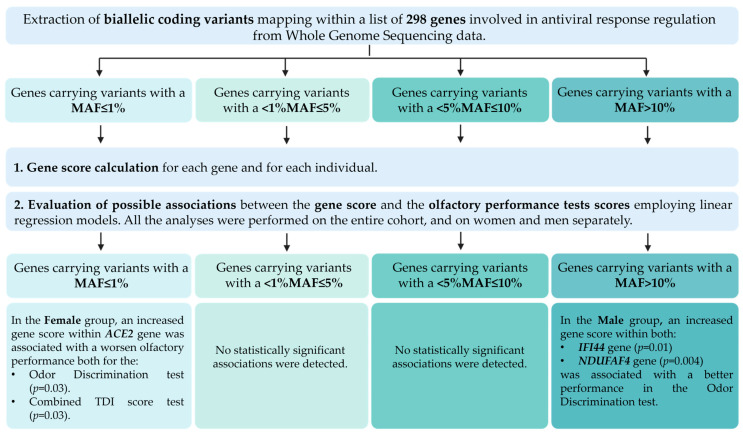
Overview of the analysis steps and results. Biallelic coding variant mapping within the list of 298 genes involved in antiviral response regulation were extracted from WGS data. The following MAF cutoffs were considered and analyzed separately: (1) MAF ≤ 1%; (2) MAF between 1% and 5%; (3) MAF between 5% and 10%; and (4) MAF > 10%. Afterwards, for each individual and for each gene, a gene score was calculated, and the association between the gene score and the orthonasal olfactory function tests results were assessed with linear regression models. For each MAF group, statistically significant results are detailed.

The association between the gene score and the psychophysically assessed olfactory performance was evaluated considering both the total score obtained by the enrolled subjects at the combined TDI test and the separate scores obtained at the threshold, discrimination, and identification subtests. Statistically significant associations were identified in the MAF ≤ 1% and MAF > 10% groups and are discussed in detail in the following paragraphs. Conversely, in the groups of MAF between 1% and 5% and MAF between 5% and 10%, no significant associations were found. A block diagram summarizing the analysis steps and the obtained results is displayed in Figure 2.

### 3.2. Association Analyses Between Rare Variants and Olfactory Performance

In order to evaluate the possible contribution of rare genetic variants in modulating the olfactory ability of patients previously affected by COVID-19, a gene score was calculated considering all biallelic variants with a MAF ≤ 1% within each gene of the antiviral response genes list.

In the female group, two statistically significant associations were detected regarding the ACE2 gene, encoding the Angiotensin-Converting Enzyme 2 (Table 3); specifically, an increased gene score within the ACE2 gene is associated with a worse olfactory performance, both for the odor discrimination (*p* = 0.03) test, and combined TDI score (*p* = 0.03).

For the male group, no statistically significant associations were identified.

### 3.3. Association Analyses Between Common Variants and Olfactory Performance

To pinpoint the role of common genetic variants in olfactory ability regulation in patients previously affected by COVID-19, a gene score was calculated considering all biallelic variants with a MAF > 10% within each gene of the analyzed list.

In males, two statistically significant associations were detected, involving *IFI44* and *NDUFAF4* genes (Table 4). In particular, an increased gene score within *IFI44* gene was associated with a better performance (*p* = 0.01) in the odor discrimination test; the same effect was observed for the *NDUFAF4* gene homozygous alternative variant carries (*p* = 0.004). No statistically significant associations were detected for females and the total cohort.

## 4. Discussion

Although most patients fully recover from SARS-CoV-2 infection, a substantial fraction of subjects develop long-lasting symptoms, which have been collectively defined as “long COVID” or “post-acute sequelae of COVID-19” (PASC) [33]. Long COVID can manifest with a broad variety of symptoms, with gastrointestinal, respiratory, and neurological ones being the most common, together with joint pain and fatigue. Additionally, chemosensory alterations, namely olfactory and gustatory dysfunction, have been reported to be considerably frequent, with a prevalence of 13–24% [34,35]. Several risk factors for persistent post-COVID-19 OD have been enumerated, such as lower severity of the acute illness, advanced age, and female gender [36]. However, despite a few studies have been conducted, full knowledge of COVID-19-related OD is far from being reached.

On the one hand, acute OD pathogenesis has been thoroughly dissected. Indeed, it has been postulated that the disruption of OE supporting cells could cause both a reduction in mucus production, with consequent impediment in the diffusion of odorant molecules to the ONs, and a decrease in glucose transport towards the ONs, which results in impairment of energy-dependent olfactory signal transduction [37]. Additionally, supporting cells are fundamental for ON cilia development and maintenance, and it has been demonstrated that sustentacular cell damage can result in the rapid retraction of cilia, ensuing complete abolishment of the signal transduction cascade [38]. In this context, ON damage is not direct but appears as a consequence of supporting cells infection, which compromises their metabolic and structural functions and, by extension, the physiological ONs activity. On the other hand, the extensive inflammatory response of the innate immune system in the OE seems to be the major driver of COIVD-19-related persistent OD. Inflammation markedly reduces the expression of genes encoding odorant receptors and other proteins involved in olfactory signal transduction through extensive chromatin reorganization due to an alteration in the neuronal nuclear architecture [39,40]. Furthermore, it has been argued that immune cell infiltration in the OE can lead to an increased apoptosis rate of epithelial cells, including ONs; if the immune response becomes chronic, it could cause considerable delay in OE regeneration, which, in some patients, may not occur at all, thus explaining the persistent OD [41]. However, not all patients develop long-lasting olfactory alterations, and, whenever they do, they present substantial variability in their onset, progression, and possible resolution, and these differences have still been poorly explored. In this light, this study focused on a cohort of 153 patients deeply characterized from a clinical and genetic point of view to identify novel host genetic factors associated with persistent OD. The availability of WGS data allowed us to select rare and common biallelic variants separately within a list of 298 genes involved in the antiviral response and calculate, for each gene, a gene score. Analyses of the accumulation of rare and common variants have increasingly been employed to deepen the knowledge of the genetic architecture of complex disorders; indeed, indeed, they particularly allow to further assess how rare and low-frequency variants contribute to the genetic landscape of these conditions [42,43,44]. Furthermore, this approach has also recently been successfully applied to study the contribution of common variants to multifactorial conditions, emphasizing how combining the contribution of both rare and common variants could highlight their additive role to the phenotype of interest [45]. As there is a growing body of literature data supporting the potential role of the host immune response in the pathophysiology of persistent OD [15,39,40,41], in this study the role of both rare and common variants was deepened, considering a list of 298 genes involved in the antiviral response regulation and, for the first time to our knowledge, was specifically aimed at the analysis of biallelic variants.

Considering MAF ≤ 1%, an increased gene score related to the *ACE2* gene was associated with worse olfactory performance in females. The *ACE2* gene encodes Angiotensin-Converting Enzyme 2, a dipeptidyl carboxydipeptidase that cleavages angiotensin I and angiotensin II into their active forms [46]. It has extensively been linked to SARS-CoV-2 infection, as viral engagement of ACE2 is essential for its entry into cells [12]. Alteration in *ACE2* expression has been associated with COVID-19 severity; in particular, virus-mediated down-regulation of *ACE2* fosters a pro-inflammatory response, which, in the acute phase of the infection, might lead to a more severe illness [47]. Additionally, its sustained dysregulation in several tissues has already been implicated in long COVID pathogenesis [48,49]. Conversely, previous studies have demonstrated how, physiologically, ACE2 has an immune regulatory function, as, by reducing angiotensin II, it decreases monocyte and endothelial cell activation [50], and, by producing Angiotensin-(1-7), it exerts powerful anti-inflammatory effects [51]. Overall, the expression pattern of *ACE2* might be critical not only for COVID-19 susceptibility but also for overall patient outcome in terms of possibility to develop PASC. In particular, in the OE, *ACE2* long-term down-regulation causes vasoconstriction, increased oxidative stress, tissue fibrosis, and inflammation, through an overproduction of pro-inflammatory cytokines, such as interleukin-1β (IL-1β), interleukin-6 (IL-6), interleukin-12 (IL-12), and interferon gamma (IFN-γ) [52]. Biopsies of the OE of patients with PASC have shown that this pro-inflammatory response is mainly mediated by T-cells and dendritic cells and directly affects sustentacular cells, thus impairing their role in ON maintenance [53]. Furthermore, it has been demonstrated that horizontal basal cells, which physiologically express *ACE2* and serve as precursor cells of ONs, also show a down-regulation of genes involved in epithelial renewal and differentiation, thus contributing to a decrease in the overall number of functional ONs in the OE [53]. In this light, despite the fact that *ACE2* dysregulation has not yet been directly associated with long-term OD, based on the above-mentioned data, it can be argued that individuals with a higher presence of rare biallelic variants in the *ACE2* gene may develop a protracted and more severe inflammatory response in the OE. The inflammatory microenvironment has already been shown to undermine the regeneration rate of the OE, which, in some patients, may not occur at all [36,41], thus leading to persistent olfactory alteration.

As concerns the MAF > 10% threshold, our study also highlighted how an increased gene score in the *IFI44* and *NDUFAF4* genes was associated with better olfactory performance. The *IFI44* gene, encoding Interferon Induced Protein 44, belongs to the subset of Interferon-stimulated genes (ISGs), and, despite it has been shown to regulate the innate immune response, little is known about its precise physiological function. ISGs have a powerful antiviral activity, being effective against positive-, negative-, and double-stranded RNA viruses, DNA viruses, and intracellular bacteria and parasites; however, recent evidence also suggests that ISGs may also negatively regulate the antiviral response, thus preventing excessive damage to the organism [54]. IFI44 is a cytoplasmatic protein that induces an antiproliferative state in cells. It has already been implicated in host response to viral infections, as it was shown that *IFI44* silencing inhibits the replication of multiple viruses, whereas its overexpression inhibits the antiviral response due to negative regulation of the interferon pathway mediated by IRF-3 and NF-κB [55]. Concerning COVID-19, a recent study has demonstrated its upregulation in patients with severe SARS-CoV-2 acute infection, together with several interferon, cytokine, and immune-related genes [56]. Additionally, it has been reported that, through the involvement of dendritic cells, it is implicated in SARS-CoV-2 replication and immune escape in patients affected by rheumatoid arthritis [57]. The *NDUFAF4* gene encodes the NADH:Ubiquinone Oxidoreductase Complex Assembly Factor 4, an assembly factor of the mitochondrial respiratory chain specifically involved in the assembly of NADH dehydrogenase (complex 1) [58]. The role of this gene in the host viral response is still poorly elucidated; however, both increased activity and inhibition of the different mitochondrial respiratory chain complexes have been shown to affect the replication and pathogenesis of many viruses [59]. Furthermore, mitochondria are involved in the production of oxygen reactive species, which are involved in the antiviral response through different mechanisms, including the IRF-3 and NF-κB signaling pathways [60]. Regarding the possible association with COVID-19, recent data show that *NDUFAF4* is differentially regulated in the immune cells of patients affected by long COVID in comparison with healthy controls [61]. Neither *IFI44* nor *NDUFAF4* have already been associated with persistent OD; however, considering literature data, it may be hypothesized that individuals with a higher accumulation of biallelic variants in both genes may present more efficient viral clearance during the acute phase of COVID-19 and therefore develop a decreased inflammatory response in the OE that thus correlates with a better outcome in olfactory performance.

Literature evidence highlights how COVID-19-related OD has shown to be related not only to increased personal and social burden, as it may provoke a loss of appetite, body weight changes, personal hygiene concerns, depression, and decrease in social and professional interactions, but also to potential harmful effects on overall individual health, as it causes an inability to recognize spoiled food with a subsequent feeling of decreased safety [62,63,64,65]. In this light, it is of paramount importance to better understand the pathophysiological mechanisms underlying this condition to identify possible therapeutic options that could improve patients’ quality of life. Many different factors have been implied in the etiology of persistent OD, and the ability of SARS-CoV-2 to cause extensive inflammatory response in the OE seems to be one of the most endorsed hypotheses. Nevertheless, it does not explain marked interindividual differences in the development of chemosensory dysfunction. In this context, our study has permitted the identification of new host genetic factors that may play a pivotal role in determining this heterogeneity. In order to additionally confirm the role of the identified genes, it would be interesting to perform a clinical follow-up for all enrolled participants to verify whether there have been further changes in their olfactory abilities and to extend the phenotypic characterization and the genetic analyses to other family members to assess possible familial aggregation.

It has to be noted that this study, conducted on a cohort of 153 Caucasian patients with mild COVID-19 while excluding severe cases, limits the generalizability of the findings to broader and more diverse populations. Despite the limited sample size, the detailed clinical and molecular characterization of the enrolled subjects allowed for a precise analysis of a homogeneous group, reducing variability linked to disease severity and ethnicity. In the future, it would be valuable to extend the analysis through the recruitment of not only Caucasian subjects but also other geographical origins in order to verify results reproducibility and possibly identify different major genetic factors that might play a prominent role in different ethnicities. A potential limitation of this study is represented by the predominance of female subjects within the cohort. Literature data show that women are more likely to develop COVID-19-related OD [66], and different hypotheses have been formulated to explain this event; for instance, it has to be considered that women present better baseline olfactory performance than men [67], thus showing greater sensitivity and a higher subjective feeling of impairment. Furthermore, immune-related X-linked genes present different expression in the immune cells of women in comparison to men [68], therefore possibly influencing the severity of the inflammatory response. In the future, it would be of paramount importance to expand the cohort of male subjects to provide a deeper characterization of the genetic factors involved in men. While focusing on 298 genes associated with antiviral response regulation excludes other potential genetic or environmental factors, this targeted approach successfully identified significant genetic associations, shedding light on the mechanisms underlying OD. In the future, it would be intriguing to further explore the possible involvement of other genetic factors through the analysis of additional genes, as, for instance, those involved in OE regeneration and maintenance. The lack of longitudinal follow-up limits the ability to track changes in olfactory function over time, but the cross-sectional design provides a clear and detailed snapshot of the relationship between genetics and olfactory performance. Lastly, although the absence of greater ethnic diversity and environmental factor analysis is a limitation, the focused scope of this study offers a basis for further research and validation in additional independent cohorts.

## 5. Conclusions

In conclusion, the results of this study contribute to shed light on the genetic architecture of long-lasting OD, offering a valuable model to investigate other forms of PASC as well and opening new possibilities to better clarify the molecular mechanisms underlying the complex physiology of the olfactory system. Indeed, as OD can be considered a multifactorial disorder that recognizes different pathophysiological mechanisms (i.e., infections, trauma, neurodegenerative disorders, aging), understanding its molecular bases could enable a more personalized and effective clinical management for affected individuals.

## Figures and Tables

**Table 1:** Demographic data and comorbidities of patients previously affected by COVID-19. The table reports the mean age of the cohort, described as mean and standard deviation (SD), and the main comorbidities, described as frequency and percentage (N (%)) for categorical variables.

Demographic Data and Comorbidities	Total, N = 153	Females, N = 111	Males, N = 42
Age			
Mean (SD)	49.2 (14.3)	51.1 (14.0)	44.2 (14.2)
Range	18.0, 90.0	18.0, 90.0	20.0, 76.0
Hypertension			
No	131 (87.3%)	95 (87.2%)	36 (87.8%)
Yes	19 (12.7%)	14 (12.8%)	5 (12.2%)
Not reported	3	2	1
Diabetes			
No	147 (97.4%)	106 (96.4%)	41 (100%)
Yes	4 (2.6%)	4 (3.6%)	0 (0%)
Not reported	2	1	1
Cardiovascular disease			
No	143 (94.7%)	104 (94.5%)	39 (95.1%)
Yes	8 (5.3%)	6 (5.5%)	2 (4.9%)
Not reported	2	1	1
Active cancer			
No	150 (99.3%)	109 (99.1%)	41 (100.0%)
Yes	1 (0.7%)	1 (0.9%)	0 (0%)
Not reported	2	1	1
Chronic respiratory disease			
No	148 (98.0%)	109 (99.1%)	39 (95.1%)
Yes	3 (2.0%)	1 (0.9%)	2 (4.9%)
Not reported	2	1	1

**Table 2:** Olfactory performance data of patients previously affected by COVID-19. The table summarizes the main results of the olfactory performance assessment data, described as mean and standard deviation (SD).

Olfactory Performance Data	Total, N = 153	Females, N = 111	Males, N = 42
Odor Threshold Test			
Mean (SD)	4.5 (2.9)	4.8 (2.8)	3.9 (3.2)
Range	0.0, 11.3	0.0, 11.3	0.0, 10.5
Odor Discrimination Test			
Mean (SD)	9.4 (2.7)	9.5 (2.6)	9.1 (2.7)
Range	2.0, 14.0	2.0, 14.0	4.0, 14.0
Odor Identification Test			
Mean (SD)	9.4 (3.2)	9.5 (3.3)	9.0 (3.0)
Range	1.0, 16.0	1.0, 16.0	3.0, 15.0
Combined TDI Score			
Mean (SD)	23.3 (7.0)	23.8 (6.9)	22.0 (7.1)
Range	4.0, 37.5	4.0, 35.5	7.0, 37.5

**Table 3:** Association between genes and olfactory performance (MAF ≤ 1.0%). The table reports the significative results of the association analyses. β Gene: effect size of the association. Adjusted *p*-value: *p*-value of the association adjusted using the Benjamini–Hochberg method.

Group	Olfactory Function Test	Gene	β Gene	Adjusted *p*-Value
Female	Odor Discrimination Test	*ACE2*	−8.21	0.03
Female	Combined TDI Score	*ACE2*	−21.70	0.03

**Table 4:** Association between genes and olfactory performance (MAF > 10%). The table reports the significative results of the association analyses. β Gene: effect size of the association. Adjusted *p*-value: *p*-value of the association adjusted using the Benjamini–Hochberg method.

Group	Olfactory Function Test	Gene	β Gene	Adjusted *p*-Value
Male	Odor Discrimination Test	*IFI44*	0.39	0.01
Male	Odor Discrimination Test	*NDUFAF4*	0.26	0.004

## Data Availability

The data underlying this article will be shared on reasonable request to the corresponding author.

## Supplementary Materials

The following supporting information can be downloaded at https://www.mdpi.com/article/10.3390/life15010056/s1, Table S1: List of 298 genes involved in antiviral response regulation.
