# Supplementary material for: Scent of COVID-19: Whole-Genome Sequencing Analysis Reveals the Role of ACE2, IFI44, and NDUFAF4 in Long-Lasting Olfactory Dysfunction
Source: Life (Basel). 2025 Jan 5;15(1):56. doi: 10.3390/life15010056 (PMC11766568; doi:10.3390/life15010056)
Supplement: Supplementary file 1 [file life-15-00056-s001.zip › life-3398132-supplementary.pdf]

Supplementary Table S1. List of 298 genes involved in antiviral response regulation. The Ingenuity Pathway Analysis software was employed to extract a list of 298 genes involved in the antiviral response regulation. The list was further manually curated in order to select only the genes described in the Online Mendelian Inheritance in Man® (OMIM®) free-access catalogue of human genes and genetic disorders. All downstream analyses have been performed on the retained genes.

ABCC9	*CCR7*	*HYAL3*	*IL6*	*OAS3*	*SPRY4*
ACE2	*CD207*	*IFI16*	*IRF1*	*OASL*	*STAT1*
ACOD1	*CD40*	*IFI27*	*IRF2*	*OPRK1*	*STAT2*
ADAR	*CD86*	*IFI44*	*IRF3*	*OSM*	*STING1*
AGBL4	*CD8A*	*IFI44L*	*IRF5*	*OTUB1*	*STK4*
AGBL5	*CGAS*	*IFI6*	*IRF7*	*OTUB2*	*TAGAP*
AIM2	*CHUK*	*IFIH1*	*IRF9*	*PARP9*	*TBK1*
APOBEC1	*CNOT7*	*IFIT1*	*ISG15*	*PCBP2*	*TICAM1*
APOBEC3A	*CREBZF*	*IFIT1B*	*ISG20*	*PCIF1*	*TICAM2*
APOBEC3B	*CXCL10*	*IFIT2*	*ITCH*	*PDE12*	*TLR2*
APOBEC3D	*CXCL12*	*IFIT3*	*ITGAX*	*PFDN6*	*TLR3*
APOBEC3F	*CXCR4*	*IFIT5*	*ITGB6*	*PLA2G10*	*TLR7*
APOBEC3G	*DDIT4*	*IFITM1*	*ITGB8*	*PLAAT4*	*TLR8*
APOBEC3H	*DDX1*	*IFITM2*	*IVNS1ABP*	*PLP1*	*TLR9*
ARMC5	*DDX21*	*IFITM3*	*JAK1*	*PLSCR1*	*TNF*
ATG16L1	*DDX3X*	*IFNA1*	*KCNJ8*	*PMAIP1*	*TNFRSF9*
ATG7	*DDX41*	*IFNA14*	*LAMTOR5*	*PPIA*	*TNFSF4*
AUP1	*DDX56*	*IFNA17*	*LCN2*	*PQBP1*	*TOMM70*
AZU1	*DDX60*	*IFNA2*	*LILRB1*	*PRF1*	*TRAF3IP1*
BANF1	*DHX15*	*IFNA4*	*LSM14A*	*PRKRA*	*TRAF3IP2*
BATF3	*DHX36*	*IFNA5*	*LYST*	*PTPN22*	*TREX1*
BCL2	*DHX58*	*IFNA7*	*MAP3K14*	*PTPRC*	*TRIM22*
BCL2L1	*DHX9*	*IFNA8*	*MAPKAPK2*	*PYCARD*	*TRIM31*
BCL3	*DICER1*	*IFNAR1*	*MAVS*	*RAB2B*	*TRIM41*
BIRC2	*DTX3L*	*IFNAR2*	*MBL2*	*RICTOR*	*TRIM44*
BIRC3	*DUOX2*	*IFNB1*	*MFN1*	*RIGI*	*TRIM5*

BNIP3	*EGFR*	*IFNE*	*MICA*	*RIPK3*	*TRIM52*
BNIP3L	*EIF2AK2*	*IFNG*	*MLKL*	*RNASE2*	*TRIM56*
BST2	*EIF2AK4*	*IFNGR1*	*MLST8*	*RNASEL*	*TRIM6*
C1R	*EXOC2*	*IFNGR2*	*MMP12*	*RNF185*	*TTC4*
C2	*EXOSC4*	*IFNK*	*MOV10*	*RNF216*	*TYK2*
C2CD4B	*EXOSC5*	*IFNL1*	*MPO*	*RPS15A*	*UNC13D*
C3	*EXT1*	*IFNL3*	*MSR1*	*RSAD2*	*URI1*
C4A	*F2RL1*	*IFNL4*	*MST1R*	*RTP4*	*USP17L2*
C4B	*FADD*	*IFNLR1*	*MX1*	*SAMHD1*	*USP21*
C4BPB	*FCGR1A*	*IFNW1*	*MX2*	*SELENOK*	*USP25*
C5	*FCN3*	*IKBKB*	*MYD88*	*SERINC3*	*USP27X*
C6	*FGR*	*IKBKG*	*NCBP1*	*SERINC5*	*VAV1*
C7	*FN1*	*IL12B*	*NCBP3*	*SETD2*	*XCL1*
C8A	*FOSL1*	*IL12RB1*	*NCR1*	*SHFL*	*XPR1*
C8G	*G3BP1*	*IL15*	*NDUFAF4*	*SIN3A*	*ZBP1*
CARD8	*GARIN5A*	*IL17C*	*NLRP1*	*SKP2*	*ZC3H12A*
CARD9	*GBP2*	*IL17RA*	*NLRP3*	*SLFN11*	*ZC3HAV1*
CC2D1A	*GBP5*	*IL1B*	*NLRP6*	*SMPD1*	*ZCCHC3*
CCL11	*GBP7*	*IL21*	*NLRP9*	*SOCS1*	*ZDHHC1*
CCL19	*GPAM*	*IL23A*	*NLRX1*	*SOCS3*	*ZDHHC11*
CCL22	*HSP90AA1*	*IL23R*	*NMI*	*SPN*	*ZMYND11*
CCL4	*HSPA8*	*IL27*	*NT5C3A*	*SPON2*	*ZNFX1*
CCL5	*HYAL1*	*IL33*	*OAS1*	*SPRY1*	
CCL8	*HYAL2*	*IL4*	*OAS2*	*SPRY2*	
